# Forward genetic studies reveal *LsAPRR2* as a key gene in regulating the green color of pericarp in bottle gourd (*Lagenaria siceraria*)

**DOI:** 10.3389/fpls.2023.1130669

**Published:** 2023-02-15

**Authors:** Yulai Huo, Gui Zhang, Wenjin Yu, Zhengguo Liu, Mujie Shen, Rongchong Zhao, Shengping Hu, Xuyang Zheng, Peng Wang, Yanjuan Yang

**Affiliations:** College of Agriculture, Guangxi University, Nanning, Guangxi, China

**Keywords:** bottle gourd, rind color, chlorophyll, APRR2, promoter variation, race

## Abstract

The fruit peel color is an important factor that affects its quality. However, genes involved in regulating pericarp color in bottle gourd (*Lagenaria siceraria*) have not been explored to date. Genetic analysis of color traits in bottle gourd peel through a genetic population of six generations demonstrated that the green color of peels is inherited as a single gene dominant trait. Combined phenotype-genotype analysis of recombinant plants using BSA-seq mapped the candidate gene to a 22.645 Kb interval at the head end of chromosome 1. We observed that the final interval contained only one gene, *LsAPRR2* (*HG_GLEAN_10010973*). Sequence and spatiotemporal expression analyses of *LsAPRR2* unraveled two nonsynonymous mutations (A→G) and (G→C) in the parental CDS sequences. Further, *LsAPRR2* expression was higher in all green-skinned bottle gourds (H16) at various stages of fruit development than in white-skinned bottle gourds (H06). Cloning and sequence comparison of the two parental *LsAPRR2* promoter regions indicated 11 bases insertion and 8 SNPs mutations in the region -991~-1033, upstream of the start codon in white bottle gourd. Proof of GUS reporting system, Genetic variation in this fragment significantly reduced the expression of *LsAPRR2* in the pericarp of white bottle gourd. In addition, we developed a tightly linked (accuracy 93.88%) InDel marker for the promoter variant segment. Overall, the current study provides a theoretical basis for comprehensive elucidation of the regulatory mechanisms underlying the determination of bottle gourd pericarp color. This would further help in the directed molecular design breeding of bottle gourd pericarp.

## Introduction

The bottle gourd [*Lagenaria siceraria (Mol.) Standl.*] (2n = 2x = 22) is native to India and Africa (Whitaker et al., 1971; Meggers et al., 1973; Erickson et al., 2005), and it is cultivated throughout China. It is also widely cultivated in tropical to temperate regions worldwide. The pericarp color is one of the most important qualities influencing consumers’ preferences. Diverse phenotypic variations in bottle gourd peel color include dark green, green, light green, white, etc. The color of the peel mainly depends on the content and form of pigments (Hao et al., 2018). However, the genetic determinants of bottle gourd peel color trait and the underlying regulatory mechanisms have not yet been studied to date.

Previously, several studies have attempted to unravel the molecular patterns of cucurbits peel color inheritance to accelerate the selective breeding process. Several studies have uncovered the genes involved in the regulation of pericarp color trait in some Cucurbitales. In melons, the gene *MELO3C003375*, which controls fruit skin color, was identified and compared among fruits with different peel color. It was observed that the CDS sequences has a 13bp insertion mutation in the fruits with yellow and white epidermal cells, which is responsible for variation in the peel color of melon (Ou, 2019). Feder et al. (2015) identified an F-box protein-encoding gene (*CmKFB*) containing a Kelch structural domain in melons. Zhao et al. (2019) Genome-wide association analysis using 635 germplasm demonstrated that the *MELO3C003375* (*APRR2*) on chromosome 4, and the *CmKFB* gene on chromosome 10 are the major regulators of green and yellow peel colors in melon. Dong et al. (2012) found that the white pericarp trait in cucumber is controlled by the cryptic single gene *w*. Further, Lun et al. (2016) reported that a Ycf54-like protein encoded by the *Csa6G133820* gene is associated with the cyclase activity in the chlorophyll biosynthesis pathway in cucumber. In addition, Liu et al. (2016) identified a candidate gene, *APRR2*, affecting the green color of the pericarp in cucumber. In white pericarp, the single base insertion of *w* gene led to the premature appearance of stop codon, which disabled the alleles involved in chlorophyll accumulation and chloroplast development. Transcriptome analysis of the transgenic lines overexpressing the *APPR2-Like* gene in tomato and a homolog of *APPR2-Like* gene in sweet pepper (*Capsicum annuum*) revealed that both genes were associated with pigment accumulation in fruit (Pan et al., 2013). A novel gene, *SG1*, was found to be highly expressed in young green tissues during a mutagenesis study in Arabidopsis. In the mutants, protoplast to chloroplast transition was delayed, thereby affecting the expression of several genes related to chloroplast development, photosynthesis, and chlorophyll biosynthesis. Overall, the study demonstrated that the protein SG1 is essential for chloroplast development in Arabidopsis (Hu et al., 2014). Identification of *Csa7G051430*, a candidate gene encoding the (ARC5) protein, plays a critical role in the chloroplast division in Arabidopsis (Zhou et al., 2015). In wax gourd, Ma et al. (2021) reported a candidate gene *Bch05G003950* (*BhAPRR2*) that controls peel color. In white peel wax gourd, a two-base (GA) deletion in the coding sequence of *BhAPRR2* with a premature stop codon affects the color of the rind. In a previous study focusing on the color of light and dark green striped watermelon, Elad et al. (2019) observed a mutation at the junction of intron 6 and exon 7, (AG→AC) in the *CLAPRR2* gene that controls peel color. The mutation resulted in a 16bp deletion in the coding region of the *CLAPRR2* gene in light green striped watermelon, triggering premature transcriptional termination, which might be linked to the light-colored rind in watermelon. In addition, Li et al. (2019) also identified a candidate gene *ClCG08G017810* (*ClCGMenG*) associated with watermelon peel color.

Variations in the chlorophyll, carotenoid, and flavonoid content are responsible for color differences between varieties (Li et al., 2007; Zhu et al., 2016). Several key transcription factors affecting the fruit’s peel color have been identified previously. MYBs are a large family of transcription factors that mainly regulate the secondary metabolite pathways such as phenylpropanoid metabolism, flavonoid biosynthesis, and anthocyanin biosynthesis in plants (Ma et al., 2021). Studies have shown that *MYB-like* transcription factors are involved in the regulation of the formation of plant anthocyanins, which play an important role in the coloration of fruit skin, flesh, and leaves (Takos et al., 2006; Azuma et al., 2008; Qing et al., 2009). The *GOLDEN2-*like (*GLK*) gene, a member of the GARP subfamily of *MYB* transcription factors, regulates chloroplast development in different plant species (Fitter et al., 2002). For example, regulation of chlorophyll expression levels by the *GOLDEN2-like* (*GLK*) transcription factor can affect the fruit color in pepper (*Capsicum annuum* L.) (Brand et al., 2014), tomato (*Solanum lycopersicum* L) (Powell et al., 2012; Nguyen et al., 2014), and Arabidopsis (*Arabidopsis thaliana*) (Fitter et al., 2002; Ni et al., 2017). Some plants require the GOLDEN2-LIKE (GLK) transcription factor for chloroplast development. Waters et al. (2009) showed that GLK proteins regulate the genes involved in light uptake and chlorophyll biosynthesis through direct interaction with promoter sequences in Arabidopsis, and it may optimize photosynthesis by coordinating the plant responses to the environment. In kiwifruit (*Actinidia chinensis*) (Li et al., 2018), and maize (*Zea mays* L) (Rossini et al., 2001), *GLK2* was found to affect chloroplast compartment size during immature fruit development. *GLK2* is shown to be a conserved and important TF that regulates chloroplast development in fleshy fruits in a variety of plants (Jia et al., 2020).

The *KNOX* family plays a key role in chlorophyll expression in plant fruits. Nadakuduti et al. (2014) showed that *TKN2* and *TKN4* belong to the class I *KNOX* family. *TKN2* and *TKN4* act upstream of *SlGLK2* and its related gene *SlAPRR2-LIKE*, thus affecting the development of chloroplasts in fruit. *TKN4* is responsible for regulating the expression of *SlGLK2*. Altogether, *TKN2* and *TKN4* regulate the expression of *GLK2* and *APRR2* (Nadakuduti et al., 2014; Jia et al., 2020).

*APRR2* is a transcription factor widely involved in chlorophyll biosynthesis in fruit. Pan et al. (2013) identified a transcription factor in tomato sharing homology with the *APRR2-LIKE* gene *APRR2-LIKE*. Overexpression of this gene in transgenic lines led to increased plastid number, area, and pigment content. While the expression levels of *APRR2-Like* were significantly higher in the leaves of transgenic tomato than in wild-type, total chlorophyll content was similar in both. Unlike *SlGLK2*, *SlAPRR2-LIKE* is thought to play a role in the maturation process (Pan et al., 2013). A putative homolog of the tomato *APRR2-like* gene in sweet pepper (*Capsicum annuum*) was associated with pigment accumulation in fruit (Pan et al., 2013). Through map-based cloning, it was found that a single base insertion in the *APRR2-Like* gene caused the termination codon to appear in advance, which was the reason for the white appearance of cucumber immature fruit (Liu et al., 2016). Recently, *APRR2* has been reported to be a candidate gene in melon and watermelon for auxiliary regulation of pigment accumulation (Elad et al., 2019). Thus, *APRR2* and *GLK2* are mutually independent TFs known to control plants’ chloroplast development (Nadakuduti et al., 2014). These studies have shown that the *APRR2-LIKE* gene plays an important role in promoting pigment accumulation and chloroplast development in plant fruits (Pan et al., 2013). Given the importance of *APRR2* in chloroplast development and pigment accumulation, it is imperative to study the potential molecular mechanism underlying the mode of action of *APRR2*.

Though several studies focusing on the peel color in the Cucurbitaceae family, the genes involved in regulating peel color in bottle gourd have not been identified to date. In the current study, we used a pair of H16 (green) and H06 (white) parent lines, and F_1_ and F_2_ generations were selected for the genetic mining of bottle gourd peel color traits. The BSA-seq combined with the phenotype-genotype analysis of recombinant plants and the sequence alignment followed by qRT-PCR were used to identify the candidate genes affecting bottle gourd peel color. Our study revealed the genetic pattern associated with green and white rind traits in bottle gourd for the first time, which is of great value and significance for studying its potential molecular regulatory mechanism. The outcomes of the current study would further help in developing genetic breeding programs for peel color traits in bottle gourd and other Cucurbitaceae family members.

## Materials and methods

### Plant material and phenotype statistics

Two bottle gourd high-generation inbred lines were selected as parents, with H06 as the white rind parent and H16 as the green rind parent; a six-generation genetic population was constructed for the genetic study of rind colour. The F_1_ generation was obtained by crossing H06 with H16 and the F_1_ generation was self-pollinated to obtain the F_2_ generation. The F_1_ was backcrossed with H06 and H16 to obtain BC_1_P_1_ and BC_1_P_2_ respectively. Parents and F_1_ planted in spring and autumn 2021 and spring 2022, BC_1_P_1_ and BC_1_P_2_ planted in spring 2022, 634 F_2_ preliminary locality population planted in autumn 2021, 5208 F_2_ finely positioned populations planted in spring 2022. The spacing within the planting rows is 0.75 m and the rows are 1.00 m. During planting, only 1 bottle gourd per plant is kept in order to ensure the healthy development of each fruit to be tested. Bottle gourds for phenotypic measurements are harvested 30 days after pollination (DAP) to ensure stable rind colour and chlorophyll content. The colour of the peel was observed with the naked eye immediately after harvest and recorded as green if it matched the colour of the green-skinned parent and as white if it matched the colour of the white-skinned parent.

### Cytological observations and pigment content assays

Microscopic observation: To observe the chloroplast morphology of the cell epidermis, well-developed fruits from both parents of 30 DAP were harvested, the thin layer of parental epidermis was carefully peeled off with a sterile scalpel, the section was placed on a slide, a drop of distilled water was added, the slide was carefully covered along one end, the light microscope was completed and then observed under a 400 × fluorescent microscope BX53 (Olympus, Japan) and photographed.

Transmission electron microscopy(TEM):To observe the ultrastructure of chloroplasts in the pericarp of two parents of 30 DAP. The fruit epidermis was quickly cut into 1-2 mm³ size with a scalpel and immediately fixed in pre-cooled electron microscope fixative (2.5% glutaraldehyde solution), the samples were rinsed with 0.1 M, pH 7.0 phosphate buffer and fixed in 1% OsO4 solution; the samples were rinsed three more times; dehydrated at room temperature and the samples were permeabilised and embedded. The samples were cut into 70-90 nm sections using a LEICA EM UC7 (LEICA, Germany). The sections were stained, dried and the chloroplast structure was observed using a transmission electron microscope Hitachi H-7650 (HITACHI, Japan).

The pericarp pigment content was measured, as described by Ma et al. (2021). After fruit harvesting, a portion of the peel with consistent color was selected, and the peel was quickly scraped, placed into liquid nitrogen, and ground to a fine powder. 0.2 g of the ground tissue was weighed into a 15 ml centrifuge tube and sealed after quickly adding 15 ml absolute ethanol. The extraction was completed by shaking at 200 rpm for 24h on a rotating instrument in a dark environment, followed by centrifugation at 6000 rpm. A microplate reader infinite M200 (TECAN, Switzerland) was used to measure the absorbance values of chlorophyll a, chlorophyll b and carotenoids at 665 nm, 649 nm and 470 nm, respectively. Each experiment was repeated three times. All experiments were performed quickly in low light conditions to reduce pigment degradation.

### DNA extraction

As described by Wilkie et al. (1997), CTAB method was used to extract genomic DNA from young leaves of parents and F_2_ generation. Detection of DNA concentration and purity using an ultramicro spectrophotometer k5800 (Kaiao, Beijing, China), and DNA quality was assessed by electrophoresis on 1.2% agarose gels.

### BSA-seq mapping approach

Bulked Segregant Analysis (BSA-seq) is a highly efficient and molecular marker-independent method for rapidly and accurately targeting genes to a compartment. It has become an effective tool for gene-targeting studies (Xu et al., 2019). We selected one green parent (H16) and one white parent (H06) for the study. Thirty extremely green individuals and 30 extremely white individuals were selected from the 634 F_2_ population for extreme admixture pool construction. Genomic DNA from young leaves was extracted, DNA samples tested for database construction, and re-sequenced using the Illumina HiSeq™ PE150 (San Diego, CA, United States) The original readings obtained from high-throughput sequencing were analyzed and converted into sequencing readings after base calls, and filtered to obtain clean readings for subsequent analysis. After filtering, two pools of extreme trait data were admixed, and two pools of parental data were constructed for association analysis. The resequenced data were compared to obtain variant information using the ZAAS_Lsic_2.0 (Xu et al., 2021) as the reference sequence. Two association analysis methods were used: the Euclidean distance (ED) association algorithm and the ΔSNP/index algorithm. Regions associated with the target traits were determined.

### Fine mapping and marker development

The kompetitive allele-specific PCR (KASP) genotyping assay exploits a competitive allele-specific PCR and fluorescence-based reporter system to efficiently identify and measure genetic variation occurring at the nucleotide level (He et al., 2014). Based on the BSA-seq results, we initially designed KASP and InDel Markers to genotype the F_2_ plants for identifying the recombinant plants (**Supplementary Table S2**). Analysis according to manufacturer ‘s instructions (LGC Genomics, Shanghai, China). In short, the kompetitive allele-specific PCR reaction contained 1.0 μL DNA (8-15 ng/μL), 1.5 μL 2 × Master Mix, 0.5 μL (10 μm) Primer mixture. Landing PCR was used for amplification. The reaction conditions were: 95°C 15min, 95°C 20s denaturation, 65 ~ 55°C annealing amplification for 1min, (10 cycles, each cycle reduced by 1.0°C), 95°C 20s denaturation, 57°C annealing amplification for 1min, (25 cycles, standard procedure), and then stored in dark conditions at 4°C. PCR products were scanned and genotyped.

The InDel-labeled PCR reaction system contained 2.0 μL genomic DNA (20-50 ng/μL), 1.0 μL each of forward and reverse primers (10 μm), 6.0 μL 2 × PCR Master Mix and 2.0 μL ddH_2_O. Reaction conditions: 95C° pre-deformation 3min, 95C° denaturation 30s, 55-60C° annealing 30s, 72C° extension 30s, (30 ~ 35 cycles, standard procedure); 72C° extension 5min followed by 4C° dark preservation. PCR amplification products were separated by 8% polyacrylamide gel electrophoresis to obtain genotyping. The primer sequence is shown in **Supplementary Table S1**.

### Candidate gene sequencing analysis

The complete coding sequence (CDS) of candidate genes was cloned and sequenced based on the reference genome (ZAAS_Lsic_2.0) (**Supplementary Table S1**). Total RNA extraction using the Eastep Super Total RNA Extraction Kit (Promega, Shanghai, China) according to manufacturer ‘s instructions. The first strand cDNA was synthesized using reverse transcriptase 5× All-In-One RT Master Mix (abm, Mississauga, Ontario, Canada). 2 × Phanta Max Master Mix (Dye Plus) PCR Premix (Vazyme, Nanjing, China) for PCR amplification. The PCR product was detected by 1.0% agarose gel electrophoresis, and the target product was purified and recovered by gel cutting. The expression vector was constructed following the manufacturer’s instructions using the CV16-Zero Background pTOPO-Blunt Cloning Kit from Aidlab (Beijing, China). The vector was transformed into Trans5α chemically compatible cells according to the manufacturer’s instructions (TransGen Biotech, Beijing, China), and monoclonal colonies were picked for sequencing. DNA fragment sequencing completed by Sangon Biotech (Shanghai) Co., Ltd. (Shanghai, China). DNA sequencing results were aligned using DNAMAN v.9 software (Lynnon Biosoft, CA, United States).

### RACE-based cloning of full-length cDNAs of candidate genes

Rapid amplification of cDNA 5’ ends (5’ RACE) was performed using the 5’-RACE Kit (Sangon, Shanghai), according to the manufacturer’s instructions. Specific primers were designed based on the validated cDNA sequences (**Supplementary Table S1**). Total RNA was extracted using the Eastep Super Total RNA Kit (Promega, Shanghai, China). Using a specific sequence in the mRNA as a binding site, the first strand cDNA was synthesized with the sequence-specific reverse transcription primer RT1/RT2 and the reagent, Reverse Transcriptase Mix (RNase H). After annealing with TdT enzyme plus 10-15 (dC) residues, (dC) residues were paired with a 5 ‘ universal adaptor primer containing the oligonucleotide sequence to perform the first round of PCR amplification using sequence-specific primer R1 as a downstream primer and first strand cDNA as template. A universal 5’ RACE outer primer containing a partial splice sequence was then used as an upstream primer. Using another specific primer R2 as a downstream primer, a cDNA fragment from the 5’ end of the gene of interest was amplified. The amplified PCR products were cut and purified by agarose gel electrophoresis, and the target products were recovered for cloning and sequencing.

Rapid amplification of cDNA 3’ ends (3’ RACE) was performed using the 3’-RACE Kit (Sangon, Shanghai), according to the manufacturer’s instructions. Specific primers were designed based on the validated cDNA sequences (**Supplementary Table S1**). First-strand cDNA was synthesized by reverse transcription using the poly (a) tail at the 3’ end of the mRNA as the 3’ adaptor for the primer binding site. Specific primer 3RF1 was used as the upstream universal primer for 3’ RACE. An 3’ RACE Outer Primer containing a partial splice sequence was used as a downstream primer. The first round of PCR amplification was performed using the first strand of cDNA as a template. The specific primer 3RF2 was then used as an upstream primer. Further, a universal primer containing a partial linker sequence, the 3’ race inner primer, was used as a downstream primer. The second round of PCR amplification was performed to amplify the DNA fragment at the 3’ end of the target gene. The amplified PCR products were purified by agarose gel electrophoresis, and the target products were recovered for cloning and sequencing. The complete sequence of the target gene was obtained by assembly and splicing of the sequence.

### RNA extraction and qRT-PCR analysis of candidate genes

To understand the expression pattern of candidate genes in different spatial and temporal contexts, quantitative real-time PCR (qRT-PCR) was performed during different stages of fruit development and in different plant organs. Total RNA was extracted from the pericarp at different stages of development and from male flowers, roots, stems, and leaves at the flowering stage. cDNA was synthesized using the Reverse Transcriptase RT Master Mix (Takara, Japan). SYBR Green real-time fluorescent quantitative PCR mixture was used for all the reactions (**Supplementary Table S1**). qRT-PCR analysis was performed using a QTOWER 2.2 qPCR instrument (Jena, Germany). The reactions mixture was prepared with 2 μL of cDNA (50 ng/μL), 0.8 μL of forward primer (10 μm), 0.8 μL of reverse primer (10 μm), 0.4 μL of ROX Reference Dye II (50×), 10 μL TB Green Premix Ex Taq II (Tli RNaseH Plus) (2×), and 6 μL of sterilized distilled water, and the final volume was made up to 20 μL followed by instant centrifugation. Preheating at 95°C for 30 s, followed by heating at 95°C for 5 s and at 60°C for 34 s for a total of 40 cycles, high-resolution melting at 95°C for 15 s, 60°C for 1 min, and 95°C for 15 s. Three replicates were performed for each sample. Relative expression was determined using the 2^−ΔΔCt^ method (Livak and Schmittgen, 2001).

### Subcellular localization of LsAPRR2

Primer design based on the complete coding sequence (CDS) of *HG_GLEAN_10010973* was obtained by RACE (**Supplementary Table S1**). The target fragment without the stop codon was amplified by PCR and cloned into the modified vector pBI221-EGFP (Li et al., 2012; Li et al., 2022). The 35S::GFP and 35S::GFP-*LsAPRR2* fusion vectors were transformed into *Agrobacterium tumefaciens* GV3101 strain. Transformed *Agrobacterium tumefaciens* cells were infiltrated into the inner epidermal cells of onion bulbs and incubated at 25°C in a light incubator. GFP signals were detected at 16h post-transformation using a laser confocal scanning microscope LEICA-TCS-SP8MP (Leica Germany), and nuclei were stained using 4 ‘, 6-diamidino-2-phenylindole (DAPI). The excitation wavelength was 488 nm.

### *LsAPRR2* promoter cloning and GUS activity analysis

The first 2000 bp of the biparental *LsAPRR2* promoter were cloned (**Supplementary Table S1**) and subsequently ligated into an expression vector (pBI121) containing a β-Glucuronidase (GUS) reporter gene (Li et al., 2022). The *Agrobacterium tumefaciens* GV3101 strain was transformed with the fusion reporter vectors as well as the empty vector. Transformed *Agrobacterium* cell suspension was injected into tobacco leaves and samples were immersed in X-Gluc buffer 7 days after injection (12 mM potassium ferricyanide, 0.3% (v/v) Triton X-100, 12 mM potassium ferrocyanide and 1 mg/ml 5-bromo-4-chloro-3-indolyl-β-D-glucuronide). The buffer was allowed to penetrate the samples under vacuum, stained overnight at 37°C, decolorized by several washes in 75% (v/v) ethanol, and photographed after complete decolorization of the chlorophyll (Koo et al., 2007).

### Phylogenetic analysis

To elucidate the relationship between the LsAPRR2 protein and its homologs, we performed a phylogenetic analysis of the LsAPRR2 homologs. The APRR2 protein sequences of some Cucurbitaceae, Solanaceae and Brassicaceae species were downloaded from NCBI (NCBI, Bethesda, MD, USA). Phylogenetic trees with 1000 Bootstrap repeats were constructed by Neighbor-joining method of MEGA 6.0 (Mega Limited, Auckland, New Zealand) software.

### Close-linked molecular marker development

Marker development was done *via* Primer Premier 5 software (Premier, Canada) (**Supplementary Table S1**). The total volume of PCR reaction was 12.0 μL, with 2.0 μL DNA (20-50 ng/μL), 1.0 μL each of forward and reverse primers (10 μm), 6.0 μL 2×PCR Master Mix, and 2.0 μL ddH_2_O. The reaction conditions were as follows: heat treatment at 95°C for 3 min, denaturation at 95°C for 30 s, annealing at 55-60°C for 30 s, extension at 72°C for 30~35 cycles; complete extension at 72°C for 5min; cool down to 4°C for storage. PCR amplicons were separated by electrophoresis using 8% native polyacrylamide gels with a voltage of 240 V and a current of 300 mA, and the program was run for 2 h. Fifty-one bottle gourd germplasm resources were validated. These germplasm resources were divided into two populations of green and white-colored fruits.

## Results

### Genetic and phenotypic analysis of epidermal color in bottle gourd

**Figure 1:**
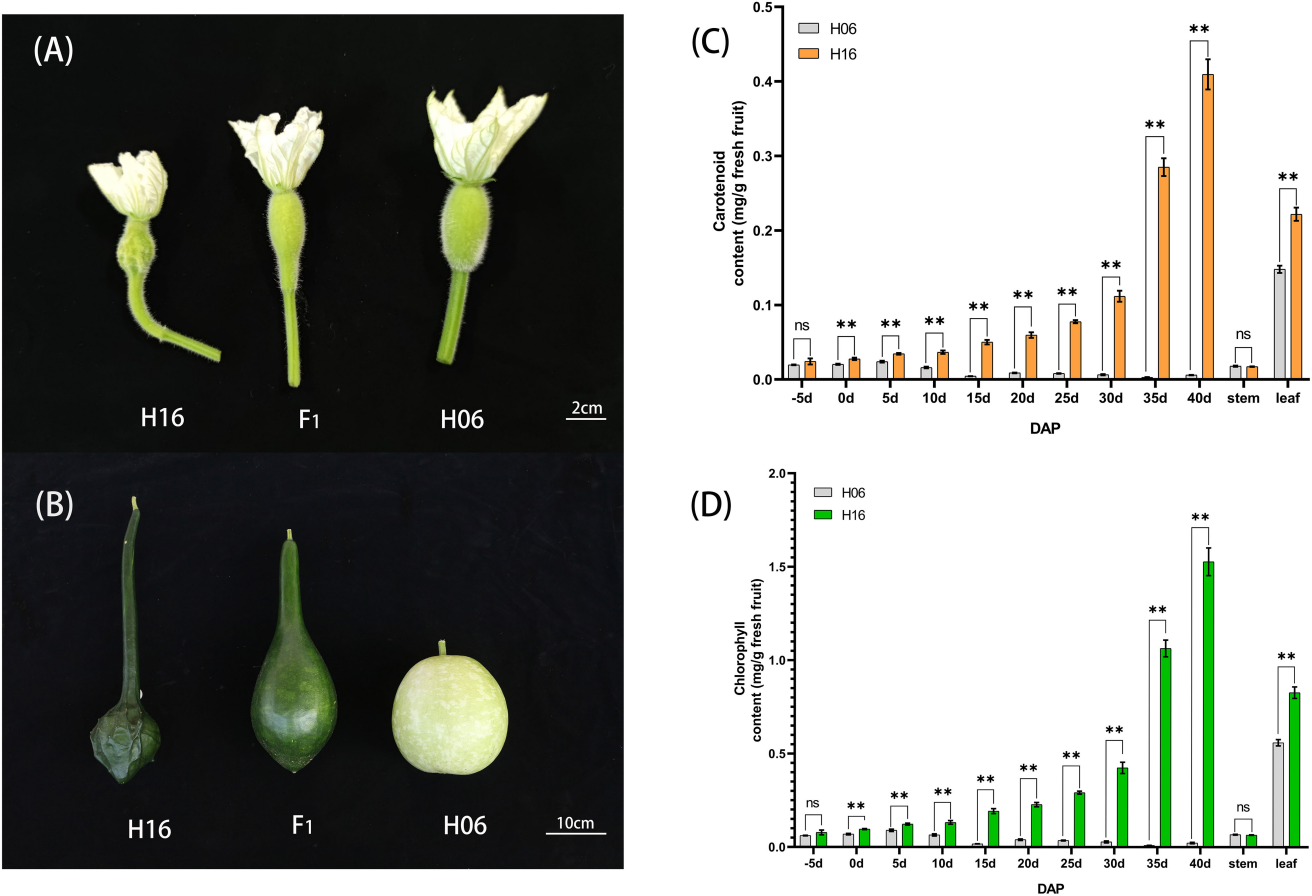
Phenotypic characteristics and pigment content analysis of gourd rind at different growth stages. **(A)** Phenotype on the day of pollination in H16, F_1_ and H06; Bar=2cm. **(B)** Phenotypes 30 days after pollination in H16, F_1_ and H06; Bar=10cm. **(C, D)** Pericarp Pigment Contents, and stem, leaf pigment contents from 5 days before pollination to 40 days after pollination for both parents. **(C)** Carotenoid content. **(D)** Chlorophyll content. *, 0.01<*P*<0.05; **, *P*<0.01. ns indicates no statistical difference.

To provide an accurate description of the changes in epidermal pigmentation pattern in bottle gourd during fruit development, we plotted chlorophyll and carotenoid profiles with growth period (**Figures 1C, D**). In the inbred combination H06×H16, the paternal line H06 had white pericarp, the maternal line H16 had green pericarp, and all F_1_ pericarp had green pericarp. Trait segregation in pericarp color occurring in the F_2_ population unraveled 149 white phenotypes and 485 green phenotypes, with a separation ratio of 1:3, respectively (χ^2^ = 0.759, ρ = 0.384). The BC_1_P_1_ plants were segregated as 67 white individuals and 66 green individuals, a distribution consistent with a 1:1 skew analysis ratio (χ^2^ = 0.008, ρ = 0.931). All the individuals harvested for BC_1_P_2_ had green epidermis (**Table 1**).

**Table 1:** Isolation of pericarp color in isolated populations of bottle gourd.

Materials	No. Of plants tested	Green: White	Expected Mendelian distribution	*χ^2^*	*P*
F_2_ BC_1_P_1_ BC_1_P_2_	634 133 105	485:149 66:67 105:0	3:1 1:1 -	0.759 0.008 -	0.384 0.931 **-**

### Chlorophyll content determination and chloroplast observation

To study the changes in pericarp pigment content during fruit development, we determined the chlorophyll and carotenoid content of the pericarp of two parents at different stages of fruit development (-5, 0, 5, 10, 15, 20, 25, 30, 35, 40DAP). The chlorophyll content of the green-skinned parent (H16) was higher than that of the white-skinned parent (H06) at all tested stages (**Figure 1D**). The chlorophyll content of green pericarp (H16) increased exponentially with fruit growth, while that of the white pericarp (H06) experienced a brief trace increase after pollination and then decreased to a lower level. Chlorophyll content measurements of both the parents’ pericarp were correlated with the visual inspection. In addition, the carotenoid content trend in both parents’ pericarp was consistent with that of chlorophyll (**Figure 1C**). The above results suggested that the H16 pericarp accumulated more pigments during growth, which prompted us to clarify further the differences in chloroplasts in H16 and H06 cells.

**Figure 2:**
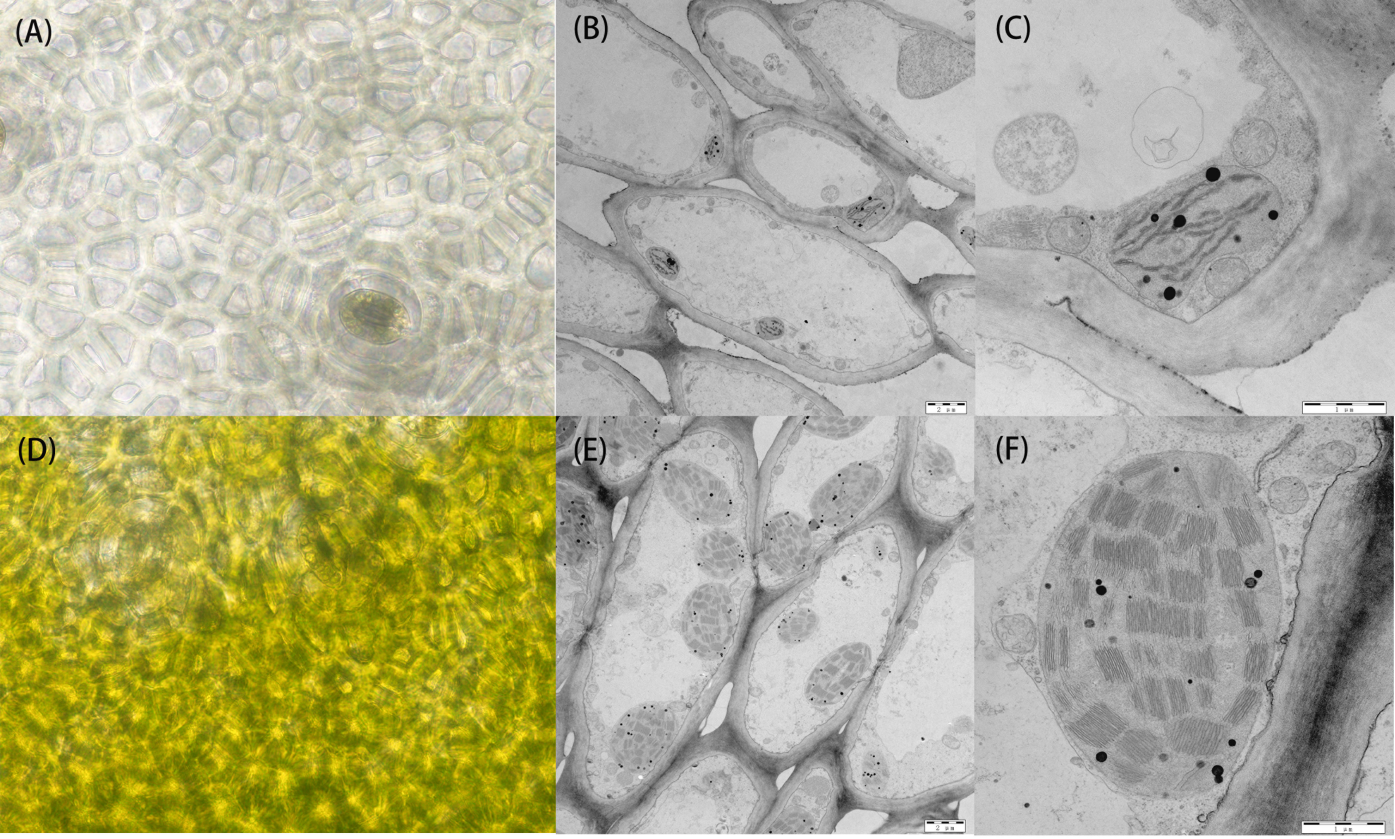
Cytology and transmission electron microscopy of 30 DAP peel from both parents. **(A)** Cytological observation of the white parental H06 pericarp. **(B, C)** transmission electron microscopy observations of H06 cells and chloroplasts, respectively. **(D)** Cytological observation of green parental H16 pericarp. **(E, F)** transmission electron microscopy observations of H16 cells and chloroplasts, respectively.

The bi-parental fruits at 30 DAP were harvested for light microscopic observation of pericarp cells. The results showed no significant difference in the size of mature cells between the two parents. In the white parent (H06), almost no chloroplasts were observed in the pericarp cells, with only visible chloroplast structures around the stomata, and the peel color was pale yellow. In contrast, in the green parent (H16), many chloroplast structures were observed in the pericarp cells, with larger and fuller chloroplasts with a dark green peel color (**Figure 2**).

To research the structural changes in chloroplasts, we used transmission electron microscopy (TEM) to analyze amphiphilic epidermal cells’ chloroplasts. It was concluded that the white parent (H06) had a low number of intracellular chloroplasts, a small chloroplast volume, and an atrophic thylakoid structure in the epidermal cells. In contrast, the green parental (H16) epidermal cell chloroplasts were more numerous, larger in size with densely packed thylakoid stacks, and plump. The chloroplasts occupied much of the cell space in the green parental epidermal cells (**Figure 2**).

### Mapping of candidate genes to the first end of chromosome 1 using BSA-seq

**Figure 3:**
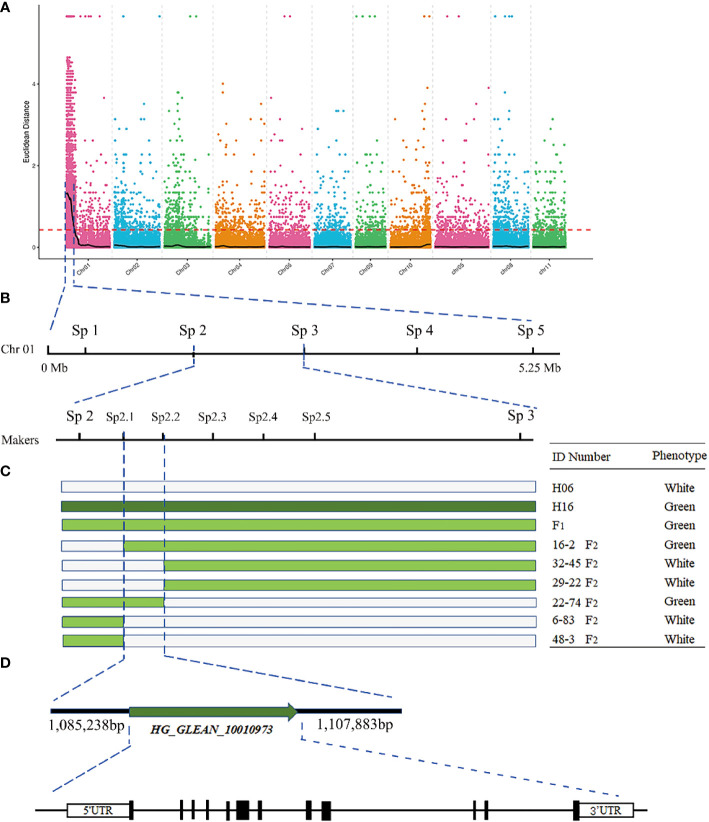
Fine mapping of the *LsAPRR2* gene in bottle gourd. **(A)** Distribution map of Euclidean distance (ED) association values on chromosomes for fruit coat color traits. **(B)** The candidate gene *LsAPRR2* is located within a 22,645bp interval between the flanking markers Sp2.1 and Sp2.2. **(C)** Genotyping of recombinant plants from F_2_, with plant numbers and phenotypic information listed on the right. **(D)** Schematic diagram of the *LsAPRR2* gene structure, with white boxes indicating the 5’UTR and 3’UTR positions of the gene and black rectangles and solid lines representing the positions where exons and introns are located, respectively.

BSA-seq-based resequencing yielded about 32,591,680 and 30,922,232 clean reads from H06 and H16 parents, respectively. In addition, 34,971,621 and 34,218,279 clean reads were obtained from the green and white mixer pools. The mean Q30 value reached 92.53 with a GC content range of 32.06 to 32.88%. The average matching efficiency of the samples to the reference genome (ZAAS_Lsic_2.0) was 99.34%, with an average coverage depth of 26.75×. The genome coverage of 99.28% (at least one base covered) was obtained in this study. Quality control indicated successful sequencing, and the data was used for subsequent mutation detection and correlation analysis. A total of 657,869 single nucleotide polymorphisms (SNPs) were detected in the four gene banks, of which 48,241 were high-quality SNPs used to calculate the SNP index between two F_2_ populations. Preliminary BSA-seq localization results were obtained with the ED and ΔSNP-index association algorithms. A confidence interval (99%) associated with the peel color trait was identified at the top end of chr01, spanning a 5.25 Mb region from 0 Mb to 5.25 Mb from the start of chr01 (**Figure 3A**). This region contained 467 genes, and SNP analysis revealed that 116 of these genes had nonsynonymous mutations.

### Fine mapping of *LsAPRR2*

To further narrow the candidate region, five pairs of KASP markers were developed at an average region of 1 Mb within the target region of 5.25 Mb initially targeted by BSA-seq. Five KASP markers were used for the combined genotype-phenotype analysis of 634 F_2_ individuals. The candidate region was between markers Sp2 and Sp3, and the region contained a physical distance of 0.94 Mb (**Figure 3B**). To further narrow the candidate region, five polymorphic InDel Markers between Sp2 and Sp3 were developed for the combined genotype-phenotype analysis of 64 recombinant plants from this region. The candidate region was mapped between markers Sp2.1 and Sp2.3, with an region physical distance of 121.37 Kb containing 11 genes. A population comprising 5208 F_2_ isolates was used to further narrow the region, which ultimately anchored the region between markers Sp2.1 and Sp2.2 at a physical distance of 22.645 Kb (**Figure 3B**). Gene annotation revealed that two annotated genes (*HG_GLEAN_10010972, HG_GLEAN_10010973*) were located within this spacer.

### RACE technique proved that *LsAPRR2* was the only gene in the interval

A partial cDNA sequence of *HG_GLEAN_10010973* was obtained from the bottle gourd reference genome (ZAAS_Lsic_2.0). Using this sequence fragment as a template, the complete sequence of *HG_GLEAN_10010973* was amplified by 5’ RACE and 3’ RACE, where the amplicon lengths were 240 bp and 68 bp, respectively. The full-length cDNA sequence of the gene was obtained by splicing three fragments and deleting the duplicate fragments. The protein sequence encoded by the cDNA of this gene consisted of 514 amino acids. After mapping this cDNA to the genome, we found that the 5’UTR of the *HG_GLEAN_10010973* gene contained *HG_GLEAN_10010972.* Therefore, we concluded that the gene *HG_GLEAN_10010972* is essentially a part of the *HG_GLEAN_10010973* gene, and the 5’ UTR sequence of *HG_GLEAN_10010973* was incorrectly annotated as a separate gene during genome assembly-based prediction. These observations highlighted that the markers Sp2.1 and Sp2.2 intervals contained only one gene, *HG_GLEAN_10010973*, which was named *LsAPRR2* in the current study (**Figure 3**).

### Sequence alignment and expression analysis of *LsAPRR2*

To analyze the sequence of *LsAPRR2*, we sequenced the full-length CDS of *LsAPRR2*, cloned from both the parents, and compared the sequencing results using DNAMAN v.9. The full-length CDS of *LsAPRR2* was 1542 bp and consisted of 12 exons. In contrast to H16, two nonsynonymous nucleotide mutations (A→G) (G→C) were detected in the CDS sequence of H06, which caused two amino acid substitutions in the H06 CDS sequence (I→M) (G→A). We used SMART (https://smart.embl.de/) for *LsAPRR2* protein prediction, which showed that the protein structure of *LsAPRR2* contains two important conserved functional domains, the REC domain (18-128 bp) and the Pfam: Myb_DNA-binding domain (279-329 bp). The two altered amino acids in the predicted results for the white epidermal parent were located in the non-conserved structural domain of the LsAPRR2 protein.

**Figure 4:**
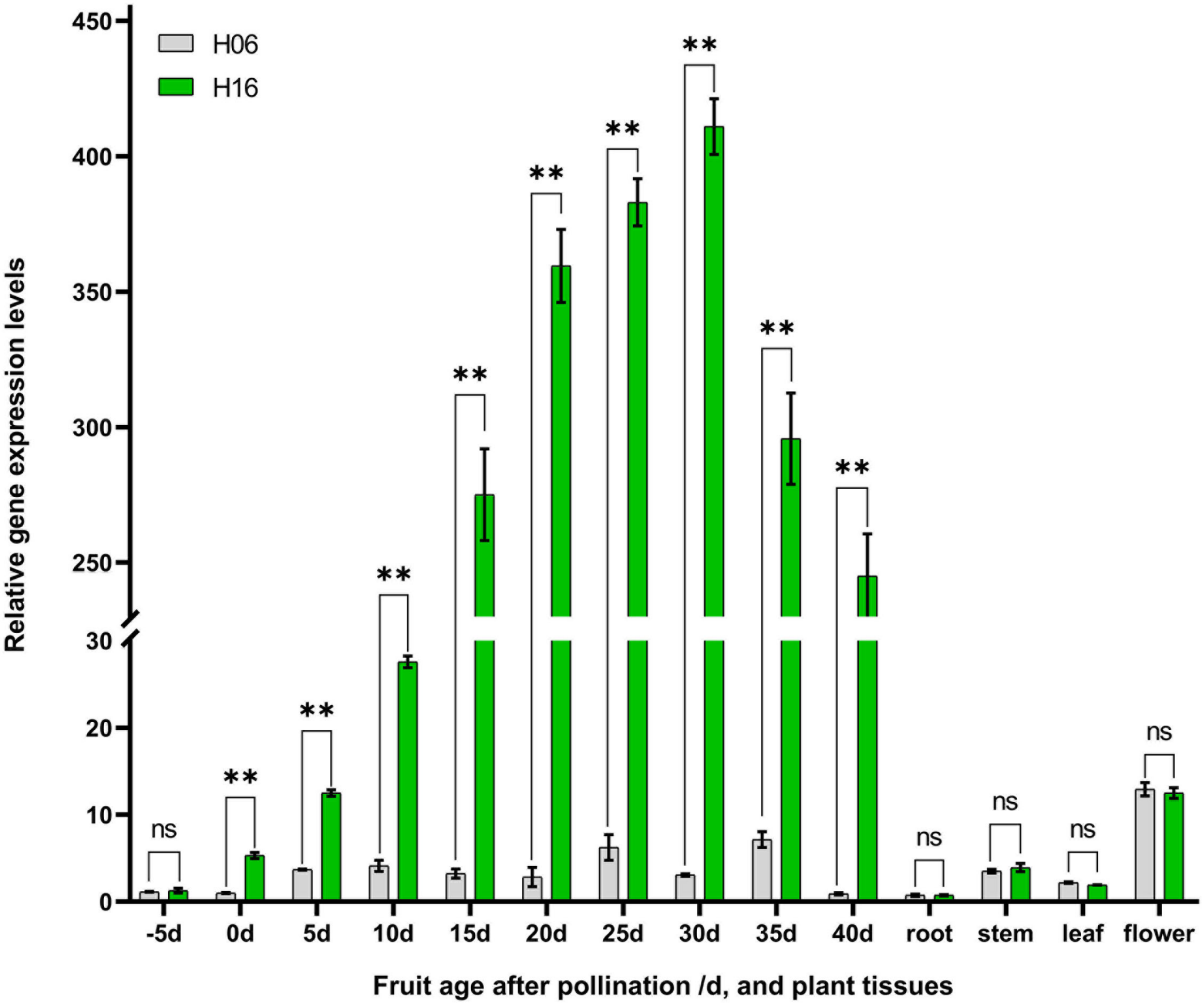
Analysis of *LsAPRR2* expression. Differential analysis of *LsAPRR2* expression in fruit epidermis from 5 days before pollination to 40 days after pollination in H06 and H16, and in root, stem, leaf, male flower. *, 0.01<*P*<0.05; **, *P*<0.01. ns indicates no statistical difference.

Spatiotemporal expression analysis of *LsAPRR2* in both parents was performed using qRT-PCR. Differential expression analysis of the pericarp (-5, 0, 5, 10, 15, 20, 25, 30, 35, 40 DAP) and different tissues (root, stem, leaf, flower) showed that expression of *LsAPRR2* was significantly higher in the pericarp of H16 than that of H06 at different times. The difference in expression reached a maximum at 30 DAP, with H16 expression reaching 133.6 times that of H06, followed by a decline in its expression. Lower *LsAPRR2* expression was observed during H06 fruit development throughout the period. The expression of *LsAPRR2* was higher in flowers than in roots, stems, and leaves (**Figure 4**).

### Subcellular localization of *LsAPRR2*

**Figure 5:**
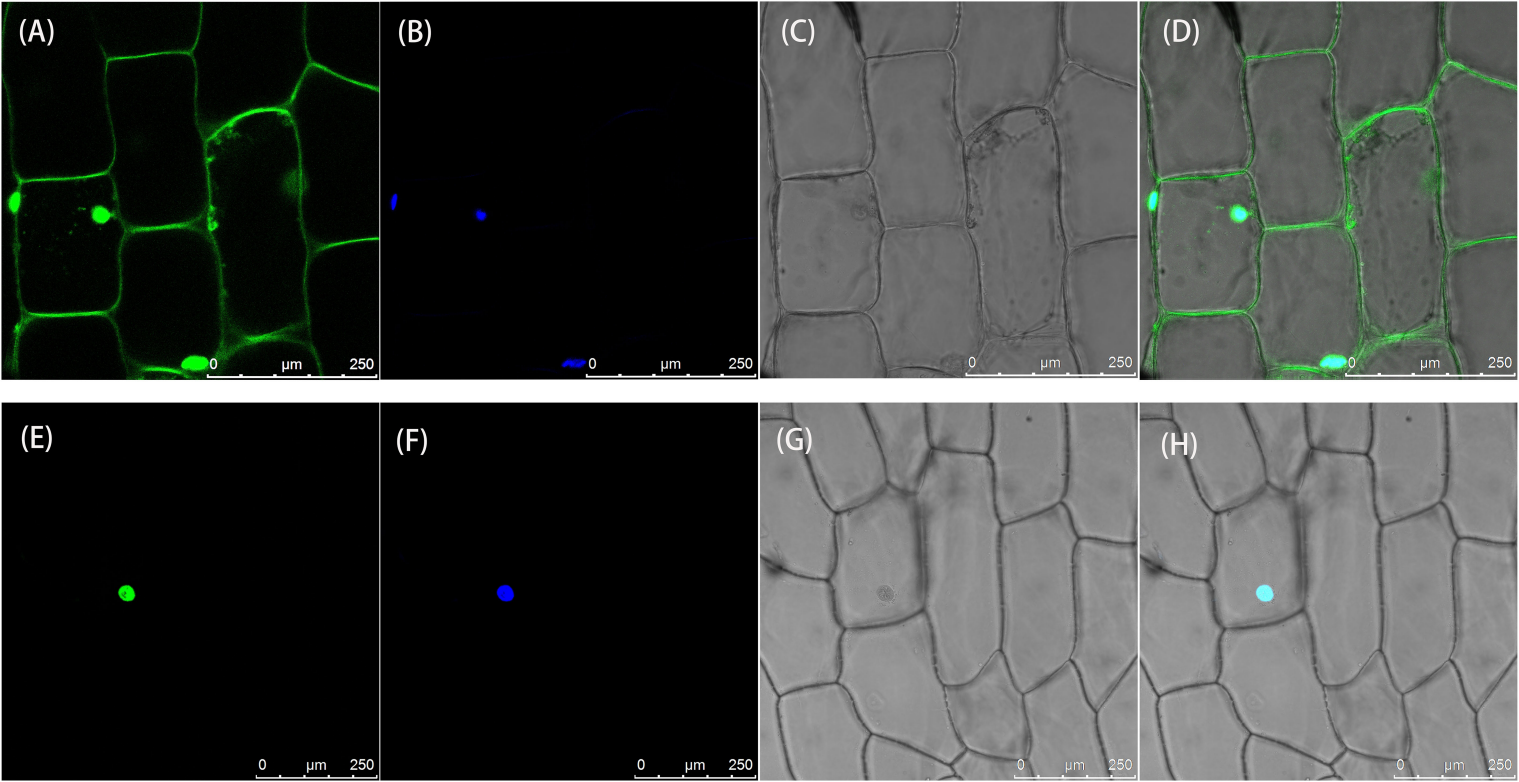
Subcellular localization of LsAPRR2. **(A–D)** Subcellular localization of the empty vector 35S::GFP. **(E–H)** Subcellular localization of 35S::GFP-LsAPRR2. **(A)** and **(E)** are the GFP fluorescence channels (green color). **(B)** and **(F)** are the DAPI fluorescence channels (glue color). **(C)** and **(G)** are bright field. **(D)** and **(H)** are overlay images.

To determine the subcellular location of the LsAPRR2 protein, 35S::GFP and 35S::GFP-*LsAPRR2* were transformed into onion inner epidermal cells. As shown in **Figure 5**, the empty vector 35S::GFP fluorescent signal was observed in whole onion cells, while the 35S::GFP-*LsAPRR2* fusion vector expression was only observed in the nuclei. The blue fluorescent signal for the DAPI stain was observed in all cell nuclei, which overlapped with the expression of the 35S::GFP-*LsAPRR2* fusion vector.

### *LsAPRR2* promoter cloning and GUS activity analysis

**Figure 6:**
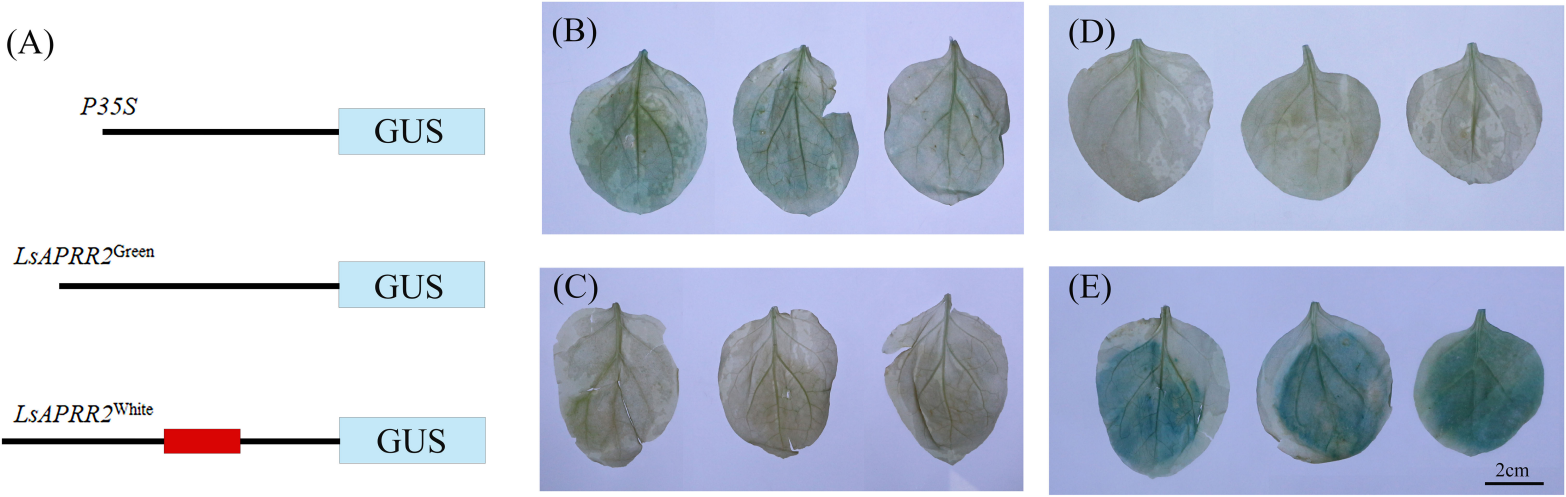
Schematic diagram of GUS fusion vector and histochemical determination of GUS activity. **(A)** Schematic diagram of the *LsAPRR2* ^Green^ and *LsAPRR2* ^White^ promoter-β-Glucuronidase (GUS) fusion vectors. **(B–E)** Histochemical assay of GUS activity in leaves of *Nicotiana benthamiana* **(B)** *LsAPRR2* ^Green^:GUS. **(C)** *LsAPRR2* ^White :^ GUS. **(D)** CK. **(E)** *P35S*:GUS.

We found that the expression of *LsAPRR2* in the rind of green-skinned bottle gourd (H16) and white-skinned bottle gourd (H06) differed significantly. To clarify all unknown variants between the two parents, we performed cloning and sequence analysis of the 2000 bp upstream region of the start codon. Sequence alignment unraveled a large number of mutations within the -991~-1033 bp promoter region upstream of the start codon (ATG). The white parent had 11 base insertions and 8 SNP mutations in this region compared to the green parent. We used PlantCARE(http://bioinformatics.psb.ugent.be/webtools/plantcare/html/)for the prediction of cis-acting elements within this region and the results showed that variants in this region had altered binding sites for TATA-box promoter elements. It has been shown that the TATA-box binding site is the core promoter element at the transcription start site around -30 bp, and alteration in this site affects the strength of the promoter (Jores et al., 2021). The GUS reporter assay showed that the 35S: GUS fusion vector had the strongest expression activity, followed by the *LsAPRR2* ^Green^: GUS fusion vector. The *LsAPRR2* ^White^: GUS fusion vector showed almost no expression (**Figure 6**). This experiment demonstrated that variants in the promoter -991~-1033 bp region affect the expression of the *LsAPRR2* gene.

### Phylogenetic analysis

**Figure 7:**
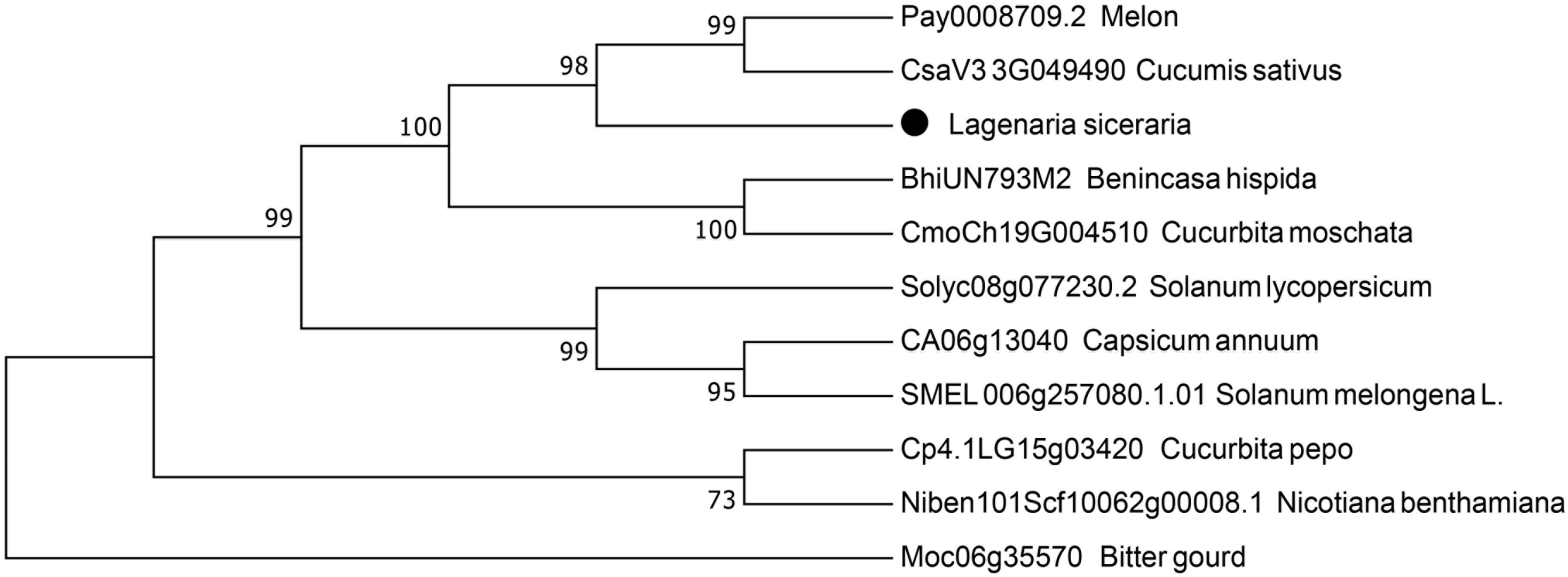
Phylogenetic analysis. Evolutionary relationship between LsAPRR2 and its cognate APRR2 protein.

We used a BLAST search in the NCBI database to obtain the sequence information, followed by phylogenetic analysis to elucidate the relationship between the LsAPRR2 protein and its homologs from different plant species (*Benincasa hispida, Melon, Cucumis sativus, Cucurbita moschata, Cucurbita pepo, Bitter gourd, Solanum lycopersicum, Capsicum annuum, Nicotiana benthamiana, Solanum melongena L.*) (**Figure 7**). The phylogenetic tree indicated that (LsAPRR2) was closely related to the Cucurbitaceae crops (*Benincasa hispida, Melon, Cucumis sativus, Cucurbita moschata*).

### Development of molecular breeding aided markers

**Figure 8:**
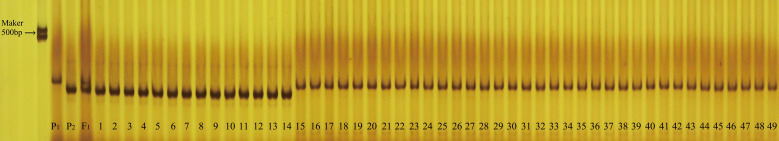
Validation of molecular breeding aid markers in 49 bottle gourd self-crossing populations. P_1_ is the white parent H06, P_2_ is the green parent H16, F_1_ is a P_1_, P_2_ cross. Numbers 1-17 are green-skinned gourds, numbers 18-49 are white-skinned gourds.

Using the sequences targeting the deletion variants in the upstream promoter of *LsAPRR2*, we developed an InDel molecular breeding-assisted marker G0913 that resulted in 362 bp and 351 bp target fragments from H06 and H16 respectively. We validated the marker against 133 BC_1_P_1_ and 5842 F_2_ populations and recorded the phenotypes and genotypes in both populations. The result showed the phenotype and genotype were 100% matche. Further, we verified 49 selfed lines of material using the G0913 marker, and the results showed that 46 of these 49 selfed lines were genotypically consistent with their phenotypes, with a concordance rate of 93.88% (**Figure 8**).

## Discussion

Peel color is one of the most important phenological qualities of bottle gourds, and thus, it is a trait of special interest for breeders. In the current study, we observed that green skin was more dominant than white skin. Further, genetic analysis of the bottle gourd peel color trait in a six-generation genetic population proved that the green peel color of bottle gourd is inherited as a single dominant gene. This is consistent with genetic models of peel color in other Cucurbitaceae crops, such as cucumber (Liu et al., 2016), wax gourd (Ma et al., 2021), and watermelon (Li et al., 2019). However, the genes that specifically regulate the peel color trait are still unknown in bottle gourd.

With the development of high-throughput sequencing technology, whole genome resequencing has become cost-effective, and data access has been more accurate and efficient. We used BSA-seq sequencing for rapid and accurate interval targeting of peel color trait-associated genes in our study. Anchoring of genes to a 1,085,238-1,107,883 bp region on chromosome 1 was completed using a novel single nucleotide polymorphism typing technique (KASP) in combination with conventional InDel markers (**Figure 3**), and the full length of the *HG_GLEAN_10010973* gene was cloned using RACE. We demonstrated that the *HG_GLEAN_10010972* gene within the candidate interval is a part of the *HG_GLEAN_10010973* 5’UTR, highlighting a previous genomic prediction error. The final interval contained only one gene, *HG_GLEAN_10010973*, which was annotated as a two-component response regulator-like APRR2 isoform ×4. We found that *APRR2-Like* was its homolog in Arabidopsis. Therefore the gene *HG_GLEAN_10010973* was named *LsAPRR2* in our study. Ou (2019) reported that the gene *MELO3C003375*, controls peel color in melon. In the melon fruits with the non-green epidermis, a 13 bp insertion was identified in the *MELO3C003375* gene, which altered the epidermal coloration of the melon. The candidate gene *APRR2* affecting green pericarp was identified in cucumber, and a single base insertion was discovered in the white pericarp gene *w*. The insertion resulted in the occurrence of a premature stop codon that disables the allele involved in chlorophyll accumulation and chloroplast development (Liu et al., 2016). A candidate gene *Bch05G003950* (*BhAPRR2*) controls peel color in wax gourd. A two-base (GA) deletion was found in the coding sequence of *BhAPRR2* in white rind winter melon resulting in a premature stop codon, affecting the peel color (Ma et al., 2021). Elad et al. (2019) found that an AG→AC mutation at the junction of intron 6 and exon 7 in the *CLAPRR2* gene, which controls watermelon peel color, resulted in a variable cut in this region, causing a 16 bp deletion in the coding region of light green striped watermelon that triggered an early termination of the transcript. Our study identified two nonsynonymous nucleotide mutations in H06 compared to H16 (A→G) (G→C). These two nucleotide variants caused a substitution of two amino acids in the protein sequence encoded by H06 compared to H16 (I→M) (G→A). Further, cloning and sequence comparison of the two parental *LsAPRR2* promoter regions revealed eleven base insertions and eight SNP mutations in the -991 to -1033 bp region in the white pericarp parent (H06). GUS assay indicated that mutations in the promoter region alter the expression of the *LsAPRR2* gene (**Figure 6**). *LsAPRR2* ^Green :^ GUS expression intensity was significantly higher than that of *LsAPRR2* ^White :^ GUS. In recent years, the *APRR2-Like* gene has been shown as a key transcription factor involved in pericarp chloroplast regulation in a variety of crops (Powell et al., 2012; Pan et al., 2013; Liu et al., 2016; Elad et al., 2019; Ma et al., 2021).

In our study, we found that the nuclear gene *LsAPRR2* influenced the color of gourd rind by regulating the development of chloroplasts. Microscopic observation of the pericarp cells showed no significant difference in size between the two parents (**Figure 2**), unlike the results described by Wang et al. (2020), where green pericarp cells were found to be smaller than white pericarp cells. The difference in pericarp cell size might be due to differences in the experimental material. Transmission electron microscopy observed that chloroplasts present in the epidermal cells of green bottle gourd were several times larger in volume than those in white epidermal cells (**Figure 2**). These findings were inconsistent with Liu et al. (2016), where the number of chloroplasts mainly caused the difference in coat color between cucumber green parent and white parent, but similar to the results by Zhu et al., 2022, in which chloroplast volume mainly affected chlorophyll accumulation leading to the changes in color. In a spatiotemporal expression analysis of *LsAPRR2* (**Figure 4**), the expression of *LsAPRR2* in the green pericarp parent (H16) was upregulated at the early stages of fruit development and gradually decreased with fruit growth after reaching 30 DAP. The white pericarp parent (H06) exhibited a small increase in *LsAPRR2* expression a few days before pollination and then remained at a low expression level. *LsAPRR2* expression in roots, stems, and leaves of the two parents was low and not significantly different (**Figure 4**), which is consistent with the pattern of variation in *APRR2* expression in fruit epidermis that has been reported in cucumber and wax gourd (Liu et al., 2016; Ma et al., 2021). Based on the chlorophyll measurement curves, we concluded that the chlorophyll content of H16 pericarp was exponentially increased after pollination and was still on the rise when *APRR2* expression was downregulated after 30 DAP (**Figure 1**). These findings were inconsistent with the studies in tomato (Pan et al., 2013) and cucumber (Liu et al., 2016), where *APRR2* expression was gradually upregulated after pollination and then decreased. Studies have shown that the gradual degradation of fruit chlorophyll during developmental stages is caused by a negative balance between synthesis and degradation of chlorophyll (Jacob-Wilk et al., 1999). However, green-skinned bottle gourds do not undergo a de-greening phase during growth. Rapid degradation of chlorophyll is a characteristic of green organs during senescence or maturation (Zhu et al., 2015). Therefore, we speculated that bottle gourd might have a different pattern of chlorophyll degradation regulation than tomato and cucumber, which undergo a de-greening phase during fruit development. We measured high chlorophyll content, whereas low expression of *APRR2* in leaves (**Figures 1**, **4**). Substantial pieces of evidence suggest that *APRR2* may be a chlorophyll regulator specifically expressed in the pericarp (Powell et al., 2012; Pan et al., 2013; Nadakuduti et al., 2014; Ma et al., 2021);. However, some recent studies have shown that *APRR2* may also be involved in color regulation in non-fruiting organs of different plants (Zhu et al., 2022) and may perform some non-color-regulated roles (Jiang et al., 2022).

Molecular marker-assisted breeding can significantly fasten the breeding process (Nadeem et al., 2018). Currently, several markers applicable in molecular marker-assisted breeding have been developed in some Cucurbitaceae crops, such as cucumber (Liu et al., 2015) and winter melon (Ma et al., 2021). We have developed an InDel molecular breeding-aided marker for a deletion variant in the upstream promoter sequence of the target gene *LsAPRR2* (**Figure 8**), which can distinguish between green and white bottle gourds (Accuracy rate of 93.88%). The lack of 100% accuracy is that there may be other genes involved in the regulation of the green epidermis of the gourd, which reduces the accuracy of the molecular markers. Based on our findings, selfed lines can be identified in the future with no variation in the *LsAPRR2* gene but with phenotypic variation to pinpoint other genes that regulate bottle gourd peel color. Also, a system of molecular markers can be generated subsequently associated with bottle gourd peel color to achieve accurate breeding for this trait.

## Data availability statement

The data that support the findings of this study have been deposited into CNGB Sequence Archive (CNSA) of China National GeneBank DataBase (CNGBdb) with accession number CNP0004006. https://db.cngb.org/search/?q=CNP0004006.

## Author contributions

YY and PW conceived and designed the experiments. YH, GZ, MS performed the experiments. GZ, MS, RZ, SH, XZ collected some data. YH analyzed the data. YH wrote the manuscript. YY, WY, ZL, PW revised the manuscript. YH, YY and PW contributed equally. All authors contributed to the article and approved the submitted version.
