# Supplementary material for: Forward genetic studies reveal LsAPRR2 as a key gene in regulating the green color of pericarp in bottle gourd (Lagenaria siceraria)
Source: Front Plant Sci. 2023 Feb 15;14:1130669. doi: 10.3389/fpls.2023.1130669 (PMC9975725; doi:10.3389/fpls.2023.1130669)
Supplement: Supplementary file 1 [file Table_1.docx]

Supplementary Material

Forward genetic studies reveal LsAPRR2 as a key gene in regulating the green color of pericarp in bottle gourd (Lagenaria siceraria)

**Yulai Huo, Gui Zhang, Wenjin Yu, Zhengguo Liu, Mujie Shen, Rongcong Zhao, Shengping Hu, Xuyang Zheng, Peng Wang* and Yanjuan Yang***

***Corresponding author:**

Yanjuan Yang （The first corresponding author）

[yjyang85@126.com](mailto:yjyang85@126.com)

Peng Wang （The sencond corresponding author）

[wangpeng@gxaas.net](mailto:840051297@qq.com)

**Table S1** Sequences of primers used in the study

| Primer ID | Annotation | Forward primer | Reverse Primer |
| --- | --- | --- | --- |
| *HG_GLEAN_10010973* | CDS amplication | ATGGTTTGCACTGCCGAC | TCAGGGAGGTTTGGTGCC |
| *LsAPRR2* | qRT-PCR analysis | TTTGCACTGCCGACGATTT | TTCCCGGTGTGTTCAAAATTG |
| *LSH3* | qRT-PCR analysis | CAAACTGCCCGTAAGTCCAC | GGCTTCTTCACTCCTCCTGT |
| Sp2.1 | InDel maker | ACCACTTGGTAAGATTGATTATGAA | AAAGTGGTTTTGTGAAGAATTTATTA |
| Sp2.2 | InDel maker | AAGCGTAAAGGGTAGGACAGAA | CTGAAAAGTTAAGACGATGAAGGA |
| Sp2.3 | InDel maker | CGAGCATGTAGTCCCTCGTT | TAGCGAGGTTGAAGGTTGGT |
| Sp2.4 | InDel maker | TTGAAGTGTTAATCCTACTCCTACC | TAAATTAATAATTTGGCTGAAAATT |
| Sp2.5 | InDel maker | TGATGTTTTTCTCTTCAAAATCCA | TTGTAGACATAACAAAACCCAAAA |
| *LsAPRR2* ^Green^ | promoter clone and GUS assay | CGCCAAGCTTGCATGCCTGCAGGAGGAGTACCTTACAAAATTGGAGTG | ATAAGGGACTGACCACCCGGGGCTTGGCATGTTTCTTATATCACTG |
| G0913 | InDel molecular assisted breeding markers | AGAATTTCAATATTTCCATCGACATCG | GAGGAATTAGGAATGAGCAACGAACA |
| RT1 | 5'RACE RT Primer | TTCGACGAATTTAGACTGCACATC |  |
| RT2 | 5'RACE RT Primer | TGAAGGACTACTTTTGACACCAGAT |  |
| 5’ adaptor Primer/R1 | 5'RACE First round amplification of cDNA | GCTGTCAACGATACGCTACGTAACGGCATGACAGTGGGGGGGGGGGGG | GATCTCCGCTTTCCCAGCTACCATGC |
| 5’RACE Outer Primer/R2 | 5'RACE Second round amplification of cDNA | GCTGTCAACGATACGCTACGTAAC | GAATTGTCACTTCCTTCTGGTTGCTCCC |
| 3’ adaptor Primer | 3'RACE RT Primer |  | GCTGTCAACGATACGCTACGTAACGGCATGACAGTGTTTTTTTTTTTTTTTTTTTT |
| 3RF1/3' RACE Outer Primer | 3'RACE First round amplification of cDNA | ATGCTGGAGGGGTGCGAGCTGATG | GCTGTCAACGATACGCTACGTAAC |
| 3RF2/3'RACE Inner Primer | 3'RACE Second round amplification of cDNA | ACATGTGGGGTCCGCCCGGTTAT | GCTACGTAACGGCATGACAGTG |

**Table S2** Primers for KASP markers

| ID | Primer X | Primer Y | Primer C |
| --- | --- | --- | --- |
| Sp1 | GAAGGTGACCAAGTTCATGCTGCCGCATCTAAACTTAGGAACTAC | GAAGGTCGGAGTCAACGGATTTGCCGCATCTAAACTTAGGAACTAT | GCTACATCTACTCTTGGCCTTTTCAC |
| Sp2 | GAAGGTGACCAAGTTCATGCTTAGACACATCTATTAGTGTTTGTTTAAAAC | GAAGGTCGGAGTCAACGGATTGATAGACACATCTATTAGTGTTTGTTTAAAAA | GTGTGTTAATTTGTTTAATTGGGCCCAC |
| Sp3 | GAAGGTGACCAAGTTCATGCTATGCATGAGGTATCGAGAACGG | GAAGGTCGGAGTCAACGGATTGAATGCATGAGGTATCGAGAACGA | CATTGTTTTTACGTTTCACGATCTCATTTTC |
| Sp4 | GAAGGTGACCAAGTTCATGCTGCCAAACCTAAAGACTAACAAAACTATTAT | GAAGGTCGGAGTCAACGGATTGCCAAACCTAAAGACTAACAAAACTATTAA | TTGGGATTTAGAATTTGTTATGGAATCTCTC |
| Sp5 | GAAGGTGACCAAGTTCATGCTGACTCAATATGCCTACCATTTTGGG | GAAGGTCGGAGTCAACGGATTGACTCAATATGCCTACCATTTTGGA | GATGTTCCCAGCTCCTCACTCTG |

**Table S3** Phenotype-genotype identification of Pericarp Color in 49 bottle gourd accessions

Resources

| Number | Variety Name | Fruit color | Genotype |
| --- | --- | --- | --- |
| 1  2  3  4  5  6  7  8  9  10  11  12  13  14  15  16  17  18  19  20  21  22  23  24  25  26  27  28  29  30  31  32  33  34  35  36  37  38  39  40  41  42  43  44  45  46  47  48  49 | H03-3a-2  H09-1b-1  H11T-1c-1  H11T-1c-2  H14c-1  H14c-2  H15-4-1  H16  H17  H20  H21  H23  H29  H07  H01  H03-3a-1  H06-1-2  H02  H03-3b  H03-3c  H04-1  H04-2  H05  H06  H06-1-1  H06-2a  H06-2b-1  H06-2b-2  H08-2  H08-3a-1  H08-3a-2  H08-3b  H09-1b-2  H10  H11T-1b-1  H12  H13  H14A  H14-B  H15-4-2  H15-4-3  H18  H19  H24  H25-1  H25-2  H26  H27  H28 | Green  Green  Green  Green  Green  Green  Green  Green  Green  Green  Green  Green  Green  Green  White  White  White  White  White  White  White  White  White  White  White  White  White  White  White  White  White  White  White  White  White  White  White  White  White  White  White  White  White  White  White  White  White  White  White | Green  Green  Green  Green  Green  Green  Green  Green  Green  Green  Green  Green  Green  Green  Green  Green  Green  White  White  White  White  White  White  White  White  White  White  White  White  White  White  White  White  White  White  White  White  White  White  White  White  White  White  White  White  White  White  White  Green |

**Table S4** Sequence alignment of H06 and H16

| Type | Sequence |
| --- | --- |
| CDS Sequence of  H06 | ATGGTTTGCACTGCCGACGATTTACAAGAATGGAAAGACTTCCCTAAGGGTCTGAGGGTTCTTCTGCTTGATCGCGACAGTCGCTCAGCTACTGAGATTAGATCAAAACTTGAGGAAATGGAGTATGTTGTTTATTCCTGCAGTGATGAGAAGGAAGCTTTGTCAGCAATTTTGAACACACCGGGAAACTTCCATGTTGCAATTCTGGAGGTGTGTGCAAGAAATTACGATGAGAGTTTTAAGTTGCTTGGAACTTCCAAGGACTTGCCAATAATAATGACTTCAGATGTTCATTGCCTAAGTACCATGATGAAGTGCATTGCACTTGGTGCAGTTGAGTTCTTGCTGAAACCACTCTCTGAGGACAAACTCAGGAATATCTGGCAGCATGTCATTCACAAGGCATTTTCCAATACTTCAAAGCCTGATGAAGATTCCGTAGCATCCTTGATGCAACTCCAATTAGAGAATGAAGACAAGAATGGAGTTCCGGAAGATATGGAAATTCTTTCTTGGATTCAGGATATTGTGTGGGAGCAACCAGAAGGAAGTGACAATTCTCAACTGAACCTGGGAGCATCTTTGCATGGTAGCTGGGAAAGCGGAGATCAAATTAACTGTTCAATGGAAACAGATTGCAGGGACAAAGATGTGCAGTCTAAATTCGTCGAAACTACTTCACATGATTTGGTTTGTGAAGGCCCCCTTCAGAAGGGCCAACCTCGATTATCTGATAAGAATAAATCTGGTGTCAAAAGTAGTCCTTCAGCTGCAGAGCACTCAATCCAAGGATCTGATGTTAACCATTCTGTTGGATCCAAAGCGAAGAAAACAAAGGTGGACTGGACCCCAGTGCTACATAGAAAATTCGTTCAAGCAGTTGAACAGTTAGGCATAGATCATGCAATTCCTTCAAAAATACTTGAGCTGATGAAAGTTGAAGGTTTGACAAGGCACAATGTTGCAAGTCATCTCCAGAAGTACAGGATGCAAAAGAAACATGTAATGCAGAGAGAAGAAAATCCAAGATGTACAATACAAACCAATCACTTGAAACCTATAATGGCATATCCTTCTTATCATCCAAACCGTGGAATATCAGTGTCTGCTGTTTATCCAACATGGAGACAGACCAATGGCCATCCAGCTAATTTCCACATGTGGGGTCCGCCCGGTTATCGCCATTGGCCACAACCAGGAATTCAGCCCTGGAATTCCTATGCTGCAGGGGTGCGAGCTGATGCATGGGGTTGCCCTGTGATACTGCCTTCTCATACTCCATATTTTTCATATCCTCGGCATGTATCAGCATCACACAATATGCATACAGTAAATAAAAGCTATGGCATGCCTCAGGGTTTATTTGATCTTCAACCAGATGAGGAGGTGGTTGACAAGATTGTAAAAGAAGCAATGAGGGAGCCATGGTCACCGCTTCCATTAGGGCTTAAACCTCCTTCTACAGAGAGTGTTCTCACAGAGCTTTCTAAGCAAGGAATCTCCACCGTCCCTCCTCAAATCAATGGCACCAAACCTCCCTGA |
| CDS Sequence of  H16 | ATGGTTTGCACTGCCGACGATTTACAAGAATGGAAAGACTTCCCTAAGGGTCTGAGGGTTCTTCTGCTTGATCGCGACAGTCGCTCAGCTACTGAGATTAGATCAAAACTTGAGGAAATGGAGTATGTTGTTTATTCCTGCAGTGATGAGAAGGAAGCTTTGTCAGCAATTTTGAACACACCGGGAAACTTCCATGTTGCAATTCTGGAGGTGTGTGCAAGAAATTACGATGAGAGTTTTAAGTTGCTTGGAACTTCCAAGGACTTGCCAATAATAATGACTTCAGATGTTCATTGCCTAAGTACCATGATGAAGTGCATTGCACTTGGTGCAGTTGAGTTCTTGCTGAAACCACTCTCTGAGGACAAACTCAGGAATATCTGGCAGCATGTCATTCACAAGGCATTTTCCAATACTTCAAAGCCTGATGAAGATTCCGTAGCATCCTTGATGCAACTCCAATTAGAGAATGAAGACAAGAATGGAGTTCCGGAAGATATGGAAATTCTTTCTTGGATTCAGGATATTGTGTGGGAGCAACCAGAAGGAAGTGACAATTCTCAACTGAACCTGGGAGCATCTTTGCATGGTAGCTGGGAAAGCGGAGATCAAATTAACTGTTCAATGGAAACAGATTGCAGGGACAAAGATGTGCAGTCTAAATTCGTCGAAACTACTTCACATGATTTGGTTTGTGAAGGCCCCCTTCAGAAGGGCCAACCTCGATTATCTGATAAGAATAAATCTGGTGTCAAAAGTAGTCCTTCAGCTGCAGAGCACTCAATCCAAGGATCTGATGTTAACCATTCTGTTGGATCCAAAGCGAAGAAAACAAAGGTGGACTGGACCCCAGTGCTACATAGAAAATTCGTTCAAGCAGTTGAACAGTTAGGCATAGATCATGCAATTCCTTCAAAAATACTTGAGCTGATGAAAGTTGAAGGTTTGACAAGGCACAATGTTGCAAGTCATCTCCAGAAGTACAGGATACAAAAGAAACATGTAATGCAGAGAGAAGAAAATCCAAGATGTACAATACAAACCAATCACTTGAAACCTATAATGGCATATCCTTCTTATCATCCAAACCGTGGAATATCAGTGTCTGCTGTTTATCCAACATGGAGACAGACCAATGGCCATCCAGCTAATTTCCACATGTGGGGTCCGCCCGGTTATCGCCATTGGCCACAACCAGGAATTCAGCCCTGGAATTCCTATGCTGGAGGGGTGCGAGCTGATGCATGGGGTTGCCCTGTGATACTGCCTTCTCATACTCCATATTTTTCATATCCTCGGCATGTATCAGCATCACACAATATGCATACAGTAAATAAAAGCTATGGCATGCCTCAGGGTTTATTTGATCTTCAACCAGATGAGGAGGTGGTTGACAAGATTGTAAAAGAAGCAATGAGGGAGCCATGGTCACCGCTTCCATTAGGGCTTAAACCTCCTTCTACAGAGAGTGTTCTCACAGAGCTTTCTAAGCAAGGAATCTCCACCGTCCCTCCTCAAATCAATGGCACCAAACCTCCCTGA |
| Protein sequence  of H06 | MVCTADDLQEWKDFPKGLRVLLLDRDSRSATEIRSKLEEMEYVVYSCSDEKEALSAILNTPGNFHVAILEVCARNYDESFKLLGTSKDLPIIMTSDVHCLSTMMKCIALGAVEFLLKPLSEDKLRNIWQHVIHKAFSNTSKPDEDSVASLMQLQLENEDKNGVPEDMEILSWIQDIVWEQPEGSDNSQLNLGASLHGSWESGDQINCSMETDCRDKDVQSKFVETTSHDLVCEGPLQKGQPRLSDKNKSGVKSSPSAAEHSIQGSDVNHSVGSKAKKTKVDWTPVLHRKFVQAVEQLGIDHAIPSKILELMKVEGLTRHNVASHLQKYRMQKKHVMQREENPRCTIQTNHLKPIMAYPSYHPNRGISVSAVYPTWRQTNGHPANFHMWGPPGYRHWPQPGIQPWNSYAAGVRADAWGCPVILPSHTPYFSYPRHVSASHNMHTVNKSYGMPQGLFDLQPDEEVVDKIVKEAMREPWSPLPLGLKPPSTESVLTELSKQGISTVPPQINGTKPP* |
| Protein sequence  of H16 | MVCTADDLQEWKDFPKGLRVLLLDRDSRSATEIRSKLEEMEYVVYSCSDEKEALSAILNTPGNFHVAILEVCARNYDESFKLLGTSKDLPIIMTSDVHCLSTMMKCIALGAVEFLLKPLSEDKLRNIWQHVIHKAFSNTSKPDEDSVASLMQLQLENEDKNGVPEDMEILSWIQDIVWEQPEGSDNSQLNLGASLHGSWESGDQINCSMETDCRDKDVQSKFVETTSHDLVCEGPLQKGQPRLSDKNKSGVKSSPSAAEHSIQGSDVNHSVGSKAKKTKVDWTPVLHRKFVQAVEQLGIDHAIPSKILELMKVEGLTRHNVASHLQKYRIQKKHVMQREENPRCTIQTNHLKPIMAYPSYHPNRGISVSAVYPTWRQTNGHPANFHMWGPPGYRHWPQPGIQPWNSYAGGVRADAWGCPVILPSHTPYFSYPRHVSASHNMHTVNKSYGMPQGLFDLQPDEEVVDKIVKEAMREPWSPLPLGLKPPSTESVLTELSKQGISTVPPQINGTKPP* |

**Table S5** Amino acid sequence information contained in phylogenetic analysis

| Name | Sequence |
| --- | --- |
| BhiUN793M2  [Benincasa hispida] | MVCTADDLQEWKDFPKGLRVLLLDRDSRSATEIRSKLEEMEYVVFSCCDEKEALSAILNTPGNFHVAILEVCARNYDESFKLLGTSKDLPIIMTSDVHCLSTMMKCIALGAVEFLLKPLSEDKLRNIWQHVIHKAFSNTSKPDEDSVASLMQLQLENENKNGVSEDMEVLSWIQDIVWEEPEGSDKSQLIMEASRQGSWESGDQMNCSIETDCRDKDVQSKFVETTSHDLVCEDPIQEGQPQLSDKNKSGVKSDPLAAENSIQGSDVNHSAGPKARKTKVDWNPQLHRKFVQAVEQLGIDHAIPSKVLELMKVEGLTRHNVASHLQKYRMQKKHVMQREENPRWSHYPRCTIQTNHLKPIMAYPSSYQPNCGISVSAVCPTWRQTNGHPPIVHTWGPPGYSHWPQRGIQPWNSYAGVRADAWGCPVMLPSHTPYFSFPQHASASHDMHTVNKSYGMPQSLCDLQPDEEVVDKIVKEAMRKPWSPLPLGLKPPSTESVLTELSRQGISTVPPQINGCRPP |
| Pay0008709.2  [Melon] | MVCTADDLQEWKDFPKGLRVLLLDRDSCSATEIRSKLEEMEYVVYSCTDEKEALSAILNTPGNFHVAILEVCARNYDEIFKLLGASKDLPIIMTSDVHCLSTMMKCIALGAVEFLLKPLSEDKLRNIWQHVIHKAYSNTSKPGEESVASLMQLQLENEDKNGVPEDMEILSWIQDIVWEQPEGSDDKSQLNLGASRQGSWESGDQMNCSMETDCKDKDVQSKFVETTSHDLVCEGPIQEGQPQLSDKKKFGVESDPLAAENSIQGTGVNQSAGSKAKKTKVKLSDTKVDWTPELHRKFVQAVEQLGIDHAIPSKILELMKVEGLTRHNIASHLQKYRMQKKHVMQREENTRWSHYPRSTLQTNHLKPIMAYPSYHPNCGISVSAVYPTWRQTNDRPPNIHVCGPFGYRHWPQPGIQPWNSYARVQADTWGCPVMPPSHAPYFSYPQLVSASQHNMHTVNKSYGMPQGLFDLQPDEEVVDKIVKEAMRQPWSPLPLGLKPPSTESVLTELSKQGISTVPPQIDGSRSP |
| CsaV3_3G049490 [Cucumis sativus] | MVCTADDLQEWKDFPKGLRVLLLDRDSFSATEIRSKLEEMEYVVYSCTDEKEASSAILNTPGNFHVAILEVCARNYDESFKLLGASKDLPIIMTSDVHCLSTMMKCIALGAVEFLLKPLSEDKLRNIWQHVIHKAYSNSSKPDEDSVASLMQFQLQNEDKNGVPEDMEILSWIQDIVWEQPEGSDDRSQLNLGASRQASWESGDQMNCSMETDCKDKDVQSKFVETTSHDLICEGPIQEGQPQLSDKKKIGVKSDPLAAENSIQELAKKHGHERGHVDWTPELHRNFVQAVEQLGIDHAIPSKILELMKVEGLTRHNIASHLQKYRMQKKHVMQREENTRWSHYPTRSTLQTNHLKPIMAYPSYHPNCGISVSAVYPTWRQTNDHPPNVHVWGPLGYRHWPQPGIQPWNSYAGVQADTWGCPVMPPSHAPYFSYPQLVSASQHNMHTVNKSYGMPQGLFDLQPDEEVVDKIVKEAMKKPWSPLPLGLKPPSTESVLTELSKKGISTVPPQIDGSRSP |
| CmoCh19G004510 [Cucurbita moschata] | MVCTVDDLQEWKDFPKGLRVLLLDRDSSSASEIRSKLEEMEYVVYSCTDEKEALSAILNTPGNFHVAILEVCARNYDESFKLLGTSMDLPIIMTSDVHCLSTMMKCIALGAVEFLLKPLSEDKLRNIWQHVLHKAFSNTPKPDEHSEASLMQLQLENKDRNEVPEEMEMLSWIQDIVWEPPEGSEKSELNLGESWQGSWESEHQMNCSMETDSREKDVYSKFVETATHDLVCEGPFQEGQPRLSGKNKSDVKSSASAAEHSIQGSDVNHSAGSKAKKSKVDWSPELHKKFVQAVEQLGIDHAIPSKILELMQVKGLTRHNVASHLQKYRMQKKHIMHREETPWRSHPRCTIQTNHLKPIMAYPSYHPNCGISPSAAFPTWRQTNALVRGPPGFCHWPRPGIHPWNSYAAGVQADAWGCPVTLPSHAPYFALPQHVLSASHNMHAVNKSYGMPQSSFDLQPDEEVVDKVVKEVMRKPCSPLPLGLKPATERVLTELSMQGISVIPPQINGSRPP |
| Cp4.1LG15g03420 [Cucurbita pepo] | MIKKHAKRAMVCTVDDLQEWKDFPKGLRVLLLDHDSSSASEIRSKLEEMEYVVYSCTDEKEALSAILNTPGNFHVAILEVCARNYDESFKLLGASMDLPIIMTSDVHCLSTMVKCIALGAVEFLLKPLSEDKLRNIWQHVLHKAFSNTPKPDEHSEASLMQLQLENKDRNEVPEEMEMLSWIQDIVWEPPEGSEKSQLNLGESLQGSWESGHQMNCSMETDSREKDVYSKFVETATHDLVCEGPFQEGQPRLSGKNKSDVKSSASAAEHSIQGSDVNHSAGSKAKKSKVDWSPELHKKFVQAVEQLGIDHAIPSKILELMKVEGLTRHNVASHLQKYRTQKKHIMHREENPWWSHPRCTIQTNHLKPIMAYPSYHPNCGISLSPVFPTWRQTNSHPGNARPPGFCHWPRPGIQPWNSYAGVQADAWGCPVTLPSHAPYFAHPQHVSSASHNMHTVNKSYGMPQSSFDLQPDEEVVDKVVKEAMRKPFSPLPLGLKPATERVLTELSMQGISIVPPQINGSRPP |
| Moc06g35570  [Bitter gourd] | MTLVISKTSSHTSFSISNRQCWRVFRMRCVVGLVTVTSLIFEVVLEALSISFFVLLFSEAYPIGKTSSSEDLAVIIVVYFVNLDQNNDLKMFEFLLMSSDLKKHAKPAMVCTADDLQGWKDFPKGLRVLLLDRDSRSTTEIRSRLEEMEYVVYSCCDEKEALSAILNTPGNFHVAILEVCTGNYDESFKLLEISKDLPIIMTSDVHCLSTMMKCIALGAVEFLLKPLSEEKLRNIWQHVIHKAFSNPSKPDEESVASLMQLQLENEDKNGVPEDMEILSWVQDIVWEQSEESEKSQLNQGASLICSWESQDQMNDSRETGCRDKETQSKLVKTTSHDLVCEEHLSESDSQPQLSGKNKSGVKSSPSVAEHSIQGSDVNHSAGLKVKKTKVDWTPDLHRKFVQAVEQLGIDHAIPSKILELMKVEGLTRHNVASHLQKYRMQKRHVIHREEIPRWPHPRCSMQFNHLKPIMAYPSSHPNCGLSVSAVYPTWRQTNGHPANVHMWGPPGYRHWPQPGIQPWNSYTGVQADAWGCPVMLPSHAPYFSNPHHVSAPHNLYTVNKSHGMPQRSFDLQPDEEMIDKVVKEAMRKPWSPLPLGLKPPSTESVLSELSRQGISTVPPHINGSRPP |
| Solyc08g077230.2 [Solanum lycopersicum] | MICIENELLGWKDFPKGLKVLLLDEDSNSAAEMKSRLEKMDYIVYSFCNESEALTAISSKSEGFHVAIVEVSAGNSDGVLRFLESAKDLPTIMTSNIHSLSTMMKCIALGAVEFLQKPLSDDKLKNIWQHVVHKAFNTRKDVSKSLEPVKDSVLSMLQLQLEMGEADDKSSNGTEPPTAVAESNTEQSSGCDKYPAPSTPQLKQGVRSVDDGDCHDHTIFSTDQDSGEHDADTKSVETTYNNSLAENNVQTSPTVQQGDIILKEDNVSSPDLKTETDIATTSRSNDCPDNSIMHSAEPSKASGPHSSNGTKSNRKKIKVDWTPELHKKFVQAVEQLGIDQAIPSRILDLMKVEGLTRHNVASHLQKYRMHRKQILPKEVERRWPNPQPIDSVQRSYYPHKPIMTFPQYHSNHVAPGGQFYPAWVTPASYPNGLQVWGSPYYPGWKPAETWHWTPRPELHADTWGSPIMSPSLGSYPPYPQNAGVYRPHGTHNRYSMLEKSFDLHPADEVIDKVVKEAITKPWLPLPLGLKAPSTESVLDELSRQGISTIPSQINDSRCRR |
| CA06g13040 [Capsicum annuum] | MVCTENDLLGWKDFPKGLRVLLLDKDSNSASDMRSRLEEMEYIVYAFCNETEALSAISSKSEVFHVAIVEVSAGNSDGGLKFLEGAKDLPTIMVSNIHSISTMMKCIALGAVEFLQKPLSDDKLRNIWQHVVHKAFHSGGKNVAESLKPVKESLLSMLELQPVKREADSENSNEAEPLTSVLENQKESPNCCDKYPAPSTPQHKQGVRSVDDGDFQDHTILSNEQDSEVHEGDTKSVETTCCDSVAETSILADSAGRLEVAITKDERDSAADQNMEDPIATCSRSNDYPADGSTRSAESNKASGLHSSSGTKANKKKMKVDWTPELHKKFVKAVEKLGIDQAIPSRILELMKVEGLTRHNIASHLQKFRMQRRQILPKEDEKRWPRPQLRDSVQRTYYYPHKPVMAFPTYHPNNAPTAGQFYPPWIPPGGYPNGAHMWGSPYYPGWQPPENWHWNPHSGLYADVWGCPVTPPSLGSCTPYLQNASGIHNRYGIIQKSVDLHPAEEVIDKVVKEAIHKPSLPLPLGLKSPSTESVLDALSKQGISAVPSRINGSRRPH |
| Niben101Scf10062g00008.1 [Nicotiana benthamiana] | MICTEDDQLLGWKDFPKGLRVLLLDEDCNSAAEMRSRLEKLDYIVYSFCNENEALSAITNKSEDFHVAIVEVTAGNSDGVLRFLESAKDLPTIIEFLQKPLSDDKLKNIWQHVVRKAFNGGEKDVPKSHKPVKESLVSMPQLQQVKSAADDKSSNETEPYTSVPDNNNEQSSGCDKYPAPSTPQLKQGVRSVDDGDCQDHTIFSTEQDSGEHDGETKSVETTFNNTIAERTVQTSPPEQQGQTIVKEENGSSPHQKVEADIATSSLSNNCPDNSSSHSAEPSKASAPHSSSGTKTNKKKMKVDWSPELHKKFVQAVEQLGIDQAIPSRILELMKVEGITRHNVASHLQKYRMHRRQILPKEVERRWPHPQPRDSVQRSYYPHHRPIMTVPPYHSNHVPAAGQLYPAWVPPASYPTGLQVWGSPYYPAWQPADSWHWNPHPGLHADTWGSPVMPPSLGSYPPYPQNGGVYRPNGMQNRYSMLEKSFDLHPAEEVIDKVVKEAITKPWLPLPLGLKRPSTESVLHELSRQGISTVPPQINGSRRS |
| SMEL_006g257080.1.01 [Solanum melongena L.] | MVCTENELLEWKDFPKGLKVLLLDKDCSSASQMRSRLQEMDYIVHTFCNENEALSAISSKSEVFHVAIVEVSDGNSDGELKFLESAKDLPTIMVSNIHSISTMMKCIALGAVEFLQKPLSDDKLRNIWQHVVHKAFHSGGKNVSESLKPVKESLLSLLELQQVKREDTNEAEPLTSVEENQKESPPCCDKYPAPSTPQQKQGVRSVDDNDLQDHTILSNEQDSGVHEGDTKSVETTCCGSIAETAVLADSARAITKEEHDSAVDQNMEDPIATCNDCPINSSIGSAHRNKASGVHSSSGTKANTKKMKVDWTPELHKKFVKAVEKLGIDQAIPSRILELMKVEGLTRHNIASHLQKFRMQRRQILPKEDERRWPRPQPRDLVQRTYYPHKPVMAFPTYHSNHASTAGQFYPAWIPPGGHPNGAHMWGSPYYPGWPPPETWHWNPQPGLYADVWGCPVTPPSLGSCTPYPQNASRFHRAEGIHNRYSSIEKSVDLHPAEEVIDKVVKEAINKPWLPLPLGLKPPSTESVLDALSKQGVSTVPPRINGSHRPH |
